# Correction of anaemia through the use of darbepoetin alfa improves chemotherapeutic outcome in a murine model of Lewis lung carcinoma

**DOI:** 10.1038/sj.bjc.6602685

**Published:** 2005-07-05

**Authors:** A M Shannon, D J Bouchier-Hayes, C M Condron, D Toomey

**Affiliations:** 1 Department of Surgery, Royal College of Surgeons in Ireland, Education and Research Centre, Smurfit Building, Beaumont Hospital, Dublin 9, Ireland

**Keywords:** erythropoietin, darbepoetin alfa, chemotherapy, anaemia, tumour oxygenation, hypoxia, drug delivery

## Abstract

Darbepoetin alfa (Aranesp®, Amgen) is a novel erythropoiesis-stimulating protein with a serum half-life longer than recombinant human erythropoietin (Epo), used in the treatment of cancer-associated anaemia. Anaemia is known to adversely affect prognosis and response to treatment in cancer patients. Solid tumours contain regions of hypoxia due to poor vascular supply and cellular compaction. Although hypoxic stress usually results in cell death, hypoxia-resistant tumour cells are genetically unstable and often acquire a drug-resistant phenotype. Increasing tumour oxygenation and perfusion during treatment could have the doubly beneficial outcome of reducing the fraction of treatment-resistant cells, while increasing drug delivery to previously hypoxic tissue. In this study, we examined the effect of darbepoetin alfa on chemotherapy sensitivity and delivery in an *in vivo* model of Lewis lung carcinoma, shown here to express the Epo receptor (EpoR). We identified that weekly darbepoetin alfa treatment, commencing 10 days before chemotherapy, resulted in a significant reduction in tumour volume compared to chemotherapy alone. This was mediated by the prevention of anaemia, a reduction in tumour hypoxia and a concomitant increase in drug delivery. Darbepoetin alfa treatment alone did not modulate the growth of the EpoR-expressing tumour cells. This study identifies an important role for darbepoetin alfa in increasing the therapeutic index of chemotherapy.

Erythropoietin (Epo) is a 30.4 kDa glycoprotein hormone that is synthesised and secreted primarily by interstitial kidney cells in response to a hypoxic stimulus or low oxygen (O_2_) tension (Krantz, 1991; Maxwell *et al*, 1993). Erythropoietin plays a vital role in the regulation of erythropoiesis, and is responsible for the maturation of Epo receptor (EpoR)-expressing erythroid progenitor cells in the bone marrow that are converted to red blood cell precursors, and subsequently to erythrocytes (Jelkmann, 1992; Lin *et al*, 1996). Darbepoetin alfa or darbepoetin (Aranesp®, Amgen) is a unique erythropoietic molecule that differs from recombinant human Epo (rHuEpo) in that it has two additional sialic acid-containing carbohydrate chains, meaning that it has a longer half-life than rHuEpo in circulation, thus requiring less-frequent dosing (Egrie and Browne, 2001).

Anaemia is a frequent clinical manifestation in the cancer patient, and may be caused by the tumour burden itself, chemotherapy drugs such as cisplatin, blood loss or diminished response to Epo (Skillings *et al*, 1993; Erslev, 2000). Although the reversal of anaemia in the cancer patient is a desirable objective, the use of Epo in this context has recently been questioned by the discovery of the EpoR on tumour cells (Westenfelder and Baranowski, 2000; Acs *et al*, 2001; Arcasoy *et al*, 2003) and on endothelial cells (Anagnostou *et al*, 1994), leading to fears that some tumours use Epo as a growth factor, or that it may promote angiogenesis (Lappin *et al*, 2002). Two separate clinical trials were designed to investigate the effects of epoetin alfa (Leyland-Jones, 2003) and beta (Henke *et al*, 2003) on cancer patients, and were terminated prematurely due to impaired disease control. However, substantial differences were found retrospectively in the epoetin alfa trial between the treatment groups, such as advanced age and lower performance status in the Epo group, which undoubtedly complicated the interpretation of the study results (Leyland-Jones, 2003). Moreover, in the epoetin beta trial (Henke *et al*, 2003), the dosing regimen raised haemoglobin levels beyond that which is recommended in normal clinical practice (>14 g dl^−1^ in women and >15 g dl^−1^ in men). Nevertheless, these results have led to controversy surrounding the role of Epo in an oncology setting.

Regions of hypoxia are a hallmark of solid tumours, meaning that O_2_ delivery to the respiring cancer cells is reduced or abolished as a result of excessive tumour cell proliferation in a confined space and an inefficient vascular structure (Dachs and Tozer, 2000; Vaupel *et al*, 2001). A number of factors associated either directly or indirectly with tumour hypoxia contribute to an overall decrease in the efficacy of chemotherapeutic agents (reviewed by Teicher *et al*, 1981; Shannon *et al*, 2003). For example, hypoxia causes cells to cycle more slowly (Amellem and Pettersen, 1991), rendering them less sensitive to cytotoxic drugs that preferentially kill rapidly proliferating cells. DNA-damaging chemotherapeutic agents such as platinum (Pt) compounds may have compromised function due to the increased activity of DNA repair enzymes under hypoxic conditions (Walker *et al*, 1994), and hypoxia also implies poor tissue permeability which affects drug delivery and distribution (Durand, 1989).

Degner and Sutherland (1988) showed that increasing blood haemoglobin concentration has a strong influence on hypoxic tissue volume. As we have discussed, the chemotherapy response of some tumours is dependent on the oxygenation status of the tumour tissue, and attempts have been made to increase low haemoglobin concentrations, thereby improving tissue oxygenation. By these standards, rHuEpo has been shown to improve tumour chemosensitivity by increasing haemoglobin concentration in various animal studies (Silver and Piver, 1999; Thews *et al*, 2001; Sigounas *et al*, 2004). The effects of darbepoetin in this context have thus far not been investigated, although it has recently been proven to be successful in increasing the therapeutic index of radiotherapy in a murine tumour model (Ning *et al*, 2005). Darbepoetin is known to have greater biological activity than rHuEpo, despite its lower affinity for the EpoR, and produces a faster rate of haemoglobin increase (Egrie and Browne, 2001; Egrie *et al*, 2003; Elliott *et al*, 2003). It is possible that this may improve response to treatment, as the longer-acting darbepoetin has been shown to result in superior responses in the prevention of chemotherapy/radiotherapy-induced anaemia, than rHuEpo (Hartley *et al*, 2003).

The chemotherapy regimen used in this study was adapted from a 12-day metronomic cisplatin and gemcitabine dosing schedule, shown to have a synergistic effect in the murine Lewis lung carcinoma (LLC) model (van Moorsel *et al*, 1999). Data suggest that cisplatin might share synergism with gemcitabine, possibly due to the multiple mechanistic interactions of the two drugs; for example, gemcitabine increases tumour Pt retention and Pt-DNA adduct levels (van Moorsel *et al*, 2000). This synergism was shown to be schedule-dependent. Recent clinical trial results indicate that gemcitabine/Pt combinations result in significant improvements in progression-free survival and overall survival in non-small-cell lung cancer (NSCLC) patients compared with other Pt-based regimens, with toxicity advantages in the cisplatin/gemcitabine combination (Natale, 2004).

This study was designed to build further on the success of metronomic chemotherapy scheduling by testing the hypothesis that darbepoetin treatment, while reducing anaemia, decreases tumour hypoxia and improves chemotherapeutic efficacy in a murine model of LLC.

## MATERIALS AND METHODS

### Cell culture

The murine LLC cell line was purchased from the ATCC and maintained in Dulbecco's modified Eagle medium (DMEM; GibcoBRL, UK) supplemented with 10% heat-inactivated FCS, 100 IU ml^−1^ penicillin and 100 *μ*g ml^−1^ streptomycin (complete medium). The K562 human chronic myelogenous leukaemia cell line was obtained from the ATCC and maintained in complete Roswell Park Memorial Institute (RPMI) 1640 medium (GibcoBRL). All cell lines were maintained at 37°C in a humidified atmosphere containing 5% CO_2_. For darbepoetin stimulation, LLC cells were growth factor deprived for 5 h by incubating in serum-free DMEM, then stimulated with 250 ng ml^−1^ darbepoetin for 20 min or left untreated as a control.

### *In vivo* study

Female 8–10-week-old C57BL/6 mice (Harlan Laboratories, UK) were used. Animals were housed in a licensed biomedical facility and had *ad libitum* access to animal chow and water. All procedures were subjected to institutional ethics review and carried out under animal licence guidelines of the Department of Health, Ireland, and according to the UKCCCR ‘Guidelines for the Welfare of Animals in Experimental Neoplasia’ (Workman *et al*, 1998). Tumours were established by subcutaneous injection of 1 × 10^6^ LLC cells in 200 *μ*l Ca^+^/Mg^+^-free PBS Dulbecco's medium (GibcoBRL) into the flank of the mice. Tumour volumes were determined by measuring the tumour width and length twice weekly (volume=0.52 × (width)^2^ × length).

### Scheduling of treatments

Chemotherapy treatment was initiated 8 days after tumour implantation, when the tumour volume was approximately 100–150 mm^3^. Day 0 was defined as the first day of intraperitoneal (i.p.) injection of chemotherapy or sterile saline (0.9% sodium chloride) injection. All drugs were administered as 100 *μ*l i.p. injections; chemotherapy drugs were diluted in saline, and darbepoetin was diluted in Ca^+^/Mg^+^-free PBS Dulbecco's medium. Mice were randomised into four experimental groups of five mice each, and received the following treatment on the days outlined in Figure 1; control (group 1) received vehicle solution; darbepoetin (group 2) received 10 *μ*g kg^−1^ darbepoetin; chemotherapy (group 3) received 3 mg kg^−1^ cisplatin (Faulding, UK) and 60 mg kg^−1^ gemcitabine (Gemzar; Eli Lilly & Co., UK); and combination chemotherapy and darbepoetin (group 4) received cisplatin, gemcitabine and darbepoetin according to the schedule in Figure 1. On the first day of chemotherapy (day 0), gemcitabine was administered 4 h prior to cisplatin.

On the final day of the study (day 12; Figure 1), mice were anaesthetised with 5% halothane (Concord Pharmaceuticals, UK) using 5 l min^−1^ O_2_. Blood samples were collected from mice in the treatment groups and from normal non-tumour-bearing mice, following a cardiac puncture. Blood was drawn up into a disposable microcuvette, and the haemoglobin (Hb) concentration was determined automatically using a Hemocue Hb 201^+^ Analyser (Hemocue Ltd, UK). Animals were then killed by cervical dislocation under an anaesthetic. The study was repeated in triplicate to provide adequate numbers of tumours for the analysis of microvessel density, hypoxia and Pt content.

### Microvessel quantification

Tumours were dissected out and flash-frozen in liquid nitrogen and stored at –80°C. Microvessel staining was carried out on 8 *μ*m frozen sections cut through the mid-section of the tumour, which were air-dried for 30 min. Sections were fixed in cold acetone (BDH Chemicals, UK), 1 : 1 acetone chloroform and acetone for 5 min each, and washed in PBS. Endogenous peroxidase activity was quenched by applying 3% hydrogen peroxide in methanol (BDH) for 10 min, and excess background was blocked using a solution of 5% normal horse serum and 1% normal goat serum (DAKO) for 20 min. Sections were incubated overnight at 4°C with 1 mg ml^−1^ anti-MECA32 (rat anti-mouse panendothelial antigen (Pharmingen, CA, USA)) diluted in blocking solution. Sections were washed in PBS, incubated in blocking solution for 10 min, then incubated with 4 *μ*g ml^−1^ horseradish peroxidase (HRP)-conjugated goat anti-rat IgG (Jackson ImmunoResearch Laboratories, PA, USA) diluted in blocking solution for 90 min at room temperature (RT). Sections were washed in PBS and antibody complexes were visualised using 3,3′diaminobenzidine tetrahydrochloride (DAB; NEN Technologies, MA, USA) for 5 min and counterstained with haematoxylin (Sigma). The sections were then washed with distilled water and mounted with Universal Mount (Research Genetics, AL, USA).

### Hypoxia quantification

Hypoxyprobe™-1 (Chemicon Europe Ltd, UK), or pimonidazole hydrochloride, is a compound which binds only to cells with a low O_2_ tension (Peters *et al*, 2004). On the final day of the study (day 12; Figure 1), animals were injected with 60 mg kg^−1^ Hypoxyprobe™-1 diluted in H_2_O for injection. After 90 min, animals were killed as before, and tumours were dissected out and stored in formalin for 24 h prior to paraffin embedding. Bound Hypoxyprobe™-1 adducts are then measured in 4 *μ*m formalin-fixed, paraffin-embedded sections cut through the midsection of the tumour, using a Hypoxyprobe™-1-specific mouse monoclonal antibody. The Animal Research Kit (ARK™, Dako) was used to visualise the antibody binding to murine tissue, as it reduces background binding to endogenous murine immunoglobulins.

After section rehydration through xylene (BDH) and a graded series of alcohols to water, the sections were washed with PBS containing 0.2% Brij 35 (Sigma) and endogenous peroxidase was quenched using a 3% solution of hydrogen peroxide in methanol for 10 min. The sections were incubated with 0.01% pronase (Sigma) for 30 min at 37°C, and rinsed in PBS-Brij. The primary antibody (Hypoxyprobe™-1 antibody: 1 : 50 dilution, immunoglobulin (Ig) concentration 70 *μ*g ml^−1^) was incubated with the appropriate volume of biotinylated F(ab′) anti-mouse Ig (ARK™), then with normal mouse serum Ig (ARK™) to block anti-mouse Ig activity. The tumour sections were incubated with the antibody complex for 15 min and rinsed in a buffer bath. Sections were incubated with HRP-conjugated streptavidin complex (ARK™) and antibody complexes were visualised as before.

### Evaluation of staining

Tumour sections from at least five tumours per treatment group were examined in a blinded manner under high-power magnification (× 200) using a Nikon Eclipse E600 light microscope, and images captured using Spot Advanced™ software (Micron Optical Co. Ltd, Ireland). For angiogenesis quantification, vessels were counted in tumour sections and the number of vessels per mm^3^ of tumour tissue was calculated. For the quantitative analysis of hypoxia, image analysis, carried out using Lucia Measurement on DS Video Version 4.82 software, determined the amount of stained hypoxic tissue (Hypoxyprobe™-1-positive) as a percentage of total area. Areas of necrotic tissue were omitted and 10 fields of view per tumour section were analysed.

### Tumour Pt determination

A relationship between Pt-DNA adduct levels and antitumour response to cisplatin in cultured cells (Terheggen *et al*, 1990) and in patient tissue (Parker *et al*, 1991; Schellens *et al*, 1996) has been demonstrated. Platinum content in the tumours was therefore measured as an indication of tumour drug/cisplatin delivery. Tumours (entire) that had been flash frozen in liquid nitrogen on day 12 (Figure 1) were weighed and digested by heating in freshly prepared Aqua Regia (HNO_3_·(3)HCl) (BDH) and made up to a known volume. A blank control was also prepared and analysed with the samples. Inductively coupled plasma mass spectrometry (ICP-MS) was carried out to quantify Pt using an Agilent 7500a ICP-MS (Centre for Microscopy and Analysis, Trinity College, Dublin, Ireland). Inductively coupled plasma mass spectrometry atomises and ionises elements in a sample and the isotopes of the elements are identified by their mass-to-charge ratio. The intensity of the specific (Pt) peak in the mass spectrum is proportional to the concentration of Pt in the original sample. The Pt concentration and original weight of the tumours were used to identify the total amount of Pt in each tumour.

### Proliferation assays

Lewis lung carcinoma cells were seeded in complete medium at a concentration of 5 × 10^3^ well^−1^ in 96-well plates and allowed to adhere overnight at 37°C in a humidified atmosphere containing 5% CO_2_. Medium was aspirated and medium containing 1% FCS alone for controls, or containing cisplatin, gemcitabine or darbepoetin at incremental concentrations, was then added to wells in triplicate, and the incubation was continued for 48 h in an aerobic or hypoxic (1% O_2_) incubator (Series II CO_2_ Incubator, ThermaForma, OH, USA). At this time, 40 *μ*l of a 3-[4,5 dimethylthiazol-2-yl]-2,5-diphenyltetrazolium bromide (MTT; Sigma) solution (0.25 mg ml^−1^ in PBS) was added directly to the media. Cells were incubated at 37°C for 1 h, culture media was aspirated and 100 *μ*l DMSO was added to each well to solubilise the purple MTT reduction product formed in viable proliferating cells. The optical density at 570 nm was recorded and the proliferation of treated cells was expressed comparatively as a percentage of untreated controls (100%). All experiments were performed in triplicate.

### EpoR Western immunoblotting

Lewis lung carcinoma cells were washed three times in cold PBS and lysis buffer (50 mM Tris-HCl, pH 7.4, 150 mM NaCl, 5 mM EDTA, 0.5% Triton-X 100, 0.5% SDS, 0.5% deoxycholic acid, 1 mM PMSF; Sigma-Aldrich, UK) was added for 1 h on ice. The cells were sheared using a syringe, and lysates cleared by centrifugation at 10 000 **g** for 30 min at 4°C. Murine anaemic spleens were used as a positive control for the EpoR. Briefly, anaemia was induced in mice using a 5 mg ml^−1^ solution of phenylhydrazine (Sigma) in PBS administered once a day for 2 days at 25 mg kg^−1^ bodyweight. Blood haemoglobin was determined 2 days later as described previously. Spleens from mice with a haemoglobin concentration <10 g dl^−1^ were flash-frozen in liquid nitrogen, lysed, briefly homogenised (Ultra-Turrax T8; IKA Labortechnik, Germany), and the lysates cleared as described previously. Total protein concentration in the lysates was determined using the bicinchoninic acid assay according to the manufacturer's instructions (Pierce, IL, USA). Equal amounts of protein were separated by 9% SDS–PAGE and transferred to nitrocellulose membranes.

Membranes were probed using an adaptation of a previously described method (Westenfelder *et al*, 1999). Briefly, blocking was carried out with 5% non-fat dry milk in Tris-buffered saline (TBS) containing 0.1% Tween-20 (TBST) for 2 h at RT. Membranes were probed for 1 h RT with 0.27 *μ*g ml^−1^ (1 : 750) goat anti-murine EpoR antibody (Santa Cruz Biotechnology, CA, USA). Membranes were washed with TBST, and incubated for 90 min with 0.5 *μ*g ml^−1^ HRP-conjugated goat anti-rabbit antibody (Dako, UK). Membranes were washed in TBST and antibody complexes were detected using enhanced chemiluminescence (Pierce, IL, USA).

### Phosphorylated STAT5 Western immunoblotting

The principal transcription factors activated by Epo are STAT5a and STAT5b (Damen *et al*, 1995; Sawyer and Penta, 1996; Barber *et al*, 2001). To detect transcription factor phosphorylation, cellular lysates were obtained from LLC cells (control and darbepoetin-stimulated). K562 lysates were used as a positive control (de Groot *et al*, 1999). Equal amounts of protein were separated by 9% SDS–PAGE and transferred to nitrocellulose membranes. Blocking was carried out with 4% BSA (Sigma) in TBST containing 50 mM NaF (Sigma) for 2 h at RT. Membranes were probed overnight at 4°C with 0.67 *μ*g ml^−1^ (1 : 300 dilution) goat anti-human pSTAT5 antibody (Santa Cruz Biotechnology). Membranes were washed with TBST, and incubated for 1.5 h with 0.05 *μ*g ml^−1^ HRP-conjugated rabbit anti-goat antibody (Dako, UK) and antibody complexes visualised as before.

The membranes that had been probed for pSTAT5 were stripped and re-probed with an antibody that recognises total STAT5 (STAT5a and STAT5b). Blocking was carried out with 5% non-fat dry milk in TBST overnight at 4°C. Membranes were probed for 1 h RT with 1 *μ*g ml^−1^ (1 : 600 dilution) rabbit anti-murine STAT5 antibody (Santa Cruz Biotechnology). Membranes were washed with TBST, incubated for 1.5 h with 0.3 *μ*g ml^−1^ HRP-conjugated goat anti-rabbit antibody (Dako, UK) and antibody complexes visualised as before.

### Statistical analysis

Statistical comparison between more than two groups was carried out by analysis of variance (ANOVA) with LSD *post hoc* correction, and between two groups by an independent-sample *t*-test, using the SPSS™ statistical software package (SPSS Inc., IL, USA). Data were expressed as mean±standard error of the mean (s.e.m.) and taken as significant where *P*<0.05.

## RESULTS

### Darbepoetin modulates chemotherapy antitumour efficacy

The administration of darbepoetin alone to tumour-bearing animals had no effect on tumour growth compared to controls (Figure 2A). Treatment with cisplatin and gemcitabine resulted in a significant decrease in tumour volume compared to controls from day 7, but, when chemotherapy and darbepoetin were combined, there was a further significant reduction in tumour volume compared with that seen in mice treated with chemotherapy alone (*P*<0.05) on days 10 and 12 (Figure 2B).

When darbepoetin was administered on the same day as chemotherapy, or 3 days before, no significant difference in tumour volume was observed (data not shown). The increase in treatment outcome was achieved only when weekly darbepoetin treatment at a concentration of 10 *μ*g kg^−1^ was initiated 10 days prior to the commencement of chemotherapy.

### Darbepoetin prevents chemotherapy-induced anaemia

Mice with a blood haemoglobin concentration of less than 10 g dl^−1^ were considered anaemic. Haemoglobin values for non-tumour-bearing mice were 13.25±0.55 g dl^−1^ (Figure 3). Tumour-bearing mice had an average blood haemoglobin concentration of 12.2±0.45 g dl^−1^ (nonanaemic) when untreated, and 13.06±0.29 g dl^−1^ when treated with darbepoetin only. There was no significant difference in haemoglobin concentration between any of these groups. The chemotherapy regimen significantly reduced haemoglobin to anaemic levels (9.1±0.39 g dl^−1^ Hb) (*P*<0.05). Weekly darbepoetin treatment beginning 10 days before chemotherapy prevented anaemia (12.1±0.34 g dl^−1^ Hb), thus maintaining haemoglobin at a ‘normal’ concentration. Darbepoetin treatment at lower doses and beginning on the same day as chemotherapy, or 3 days before, did not result in the correction of chemotherapy-induced anaemia (data not shown).

### Darbepoetin has no effect on tumour angiogenesis

The extent of vascularisation in tumour tissue from animals in the treatment groups was measured by the immunohistochemical detection of panendothelial (MECA32) antigen (Figure 4). Statistical analysis showed that darbepoetin treatment did not influence tumour microvessel formation.

### Darbepoetin reduces anaemia-induced tumour hypoxia

Hypoxyprobe™-1 staining showed that tumours from nonanaemic mice in the control and darbepoetin-treated groups contained hypoxic regions of between 4.3 and 5.4% of total tumour area (Figure 5). Darbepoetin treatment alone increased haemoglobin by ∼1 g dl^−1^; therefore, we did not expect a significant decrease in hypoxia in the darbepoetin-treated tumours. The chemotherapy-treated tumours from anaemic mice contained significantly higher levels of Hypoxyprobe™-1 staining (11±1.7% of tumour area) and, therefore, the lowest level of oxygenation (*P*<0.05). However, when darbepoetin treatment began 10 days before chemotherapy, preventing anaemia, there was a significant reduction in the percentage of hypoxic tissue to 3±0.4% of total tumour area, compared to tumours treated with chemotherapy alone.

### Darbepoetin increases tumour cisplatin delivery

Total Pt levels in the tumours were measured using ICP-MS as an indication of tumour cisplatin concentration. Tumours in the combination chemotherapy and darbepoetin-treated group contained significantly higher amounts (*P*<0.05) of Pt than tumours in the group treated with chemotherapy only (Figure 6).

### Chemotherapy efficacy is increased under aerobic conditions

The cytotoxic effects of cisplatin (Figure 7A) and gemcitabine (Figure 7B) were assessed *in vitro* under both aerobic and hypoxic growth conditions to demonstrate the sensitivity of cells to chemotherapy under different environmental conditions. Both cisplatin and gemcitabine reduced tumour cell proliferation to 30–40% of controls under aerobic conditions (*P*<0.05). Under hypoxic conditions, tumour cell proliferation was reduced; however, the treatment of cells with either chemotherapy had no further effect on hypoxic cells.

### Lewis lung carcinoma cells express the EpoR

Lewis lung carcinoma cells were examined for EpoR protein expression by Western blot analysis, using a murine anaemic spleen as a positive control, as anaemia stimulates the accumulation of EpoR-expressing erythroid progenitor cells in the spleen (Youssoufian *et al*, 1993). There was specific binding of the antibody directed against the murine EpoR to a 72 kDa band on LLC cells and by anaemic spleen cells (Figure 8).

### Darbepoetin does not stimulate LLC cell proliferation

To investigate whether the EpoR on LLC cells was functional, we determined the effect of *in vitro* darbepoetin treatment on tumour cell proliferation using the MTT assay. Given reports showing that Epo may be used as a survival factor by some cells under hypoxic conditions (Sinor and Greenberg, 2000), we carried out the experiment under both aerobic and hypoxic (1% O_2_) conditions. The results show that hypoxic conditions significantly reduced cellular proliferation compared to aerobic controls (Figure 9). Treatment with darbepoetin did not influence the proliferation rate of the EpoR-expressing tumour cells under either aerobic or hypoxic growth conditions, indicating that the receptor may be nonfunctional on these cells.

### Darbepoetin does not induce phosphorylation of STAT5 in LLC cells

To further investigate the functionality of the EpoR, we looked at phosphorylation of STAT5. STAT5 phosphorylation does not occur in response to darbepoetin treatment in LLC cells (Figure 10). The antibody used in this study has previously been demonstrated to bind phosphorylated tyrosine of STAT5a of murine origin (Goleva *et al*, 2002). Blots were subsequently stripped and probed using an antibody that recognises total STAT5 to verify equal loading of samples. STAT5 consists of STAT5a (94 kDa) and STAT5b (96 kDa), and therefore appears as a double band.

## DISCUSSION

This study provides evidence that weekly darbepoetin supplementation indirectly increases the therapeutic index of a metronomic cisplatin and gemcitabine chemotherapy schedule in a murine model of LLC. The observed increase in treatment outcome was dependent on the prevention of anaemia, which resulted in decreased tumour hypoxia. The hypothesis that Epo treatment to maintain a normal haematocrit enhances chemotherapy antitumour efficacy has previously been investigated. Increased cisplatin-induced tumour regression was found in combination with rHuEpo treatment (Silver and Piver, 1999). Another group artificially induced anaemia by carboplatin administration and used rHuEpo to correct anaemia and increase drug sensitivity (Thews *et al*, 2001). Sigounas *et al* (2004) showed, also in a LLC murine model, that rHuEpo synergised with cisplatin to further suppress tumour growth. However, the cisplatin regime used in this study did not induce anaemia, so the enhancement of treatment efficacy in this instance was not related to the prevention of anaemia. The investigators hypothesised that the results reflected improved oxygenation of the hypoxic tumour tissue, as a result of the increase in O_2_ availability due to rHuEpo. To our knowledge, we are the first to prove, through the measurement of pimonidazole adducts, that darbepoetin decreases tumour hypoxia in chemotherapy-treated tumours compared with tumours from anaemic mice treated with chemotherapy alone. We show that, even with a small decrease in tumour tissue hypoxia, an improvement in outcome can be achieved, confirming the negative impact of hypoxia on prognosis.

Ning *et al* (2005) recently demonstrated that the correction of anaemia in tumour-bearing mice, by darbepoetin, increased tumour oxygenation and subsequently sensitivity towards a 5-day course of radiotherapy. The use of recombinant Epo proteins to restore anaemia-induced reduction in radiosensitivity is well documented (Thews *et al*, 1998; Stuben *et al*, 2003). Interestingly however, this paper provides information that radiotherapy efficacy is augmented, even in the absence of the correction of anaemia or tumour hypoxia. The tumour cells employed in that experiment did not express the EpoR, ruling out a direct antitumour effect. In the present study, we provide the information that, although LLC cells express the EpoR, darbepoetin treatment does not stimulate proliferation of the cells or result in STAT5 phosphorylation. As the EpoR is redundant on the tumour cells, this verifies that the correction of anaemia is the explanation for the increased therapeutic outcome. This paper supports the reports from long-term follow-up in darbepoetin clinical trials that indicate no tumour-promoting or detrimental effect of darbepoetin on survival in patients with cancer (Vansteenkiste *et al*, 2002; Hedenus *et al*, 2003).

Darbepoetin did not affect the number of tumour microvessels in our model, but, as it served to reduce tumour hypoxia in chemotherapy-treated tumours, downregulation of hypoxia-inducible vascular endothelial growth factor (VEGF) expression and vascular permeability could occur (Shweiki *et al*, 1992; Dvorak *et al*, 1999). Permeable tumour vessels in a disorganised vasculature lead to interstitial fluid pressure, impairing drug delivery to tumours (Netti *et al*, 1999). Darbepoetin could therefore function to maintain normal vasculature integrity, explaining the increased Pt content in the combination darbepoetin and chemotherapy-treated tumours, compared to tumours treated with chemotherapy alone.

Cisplatin (Koch *et al*, 2003) and gemcitabine (Yokoi and Fidler, 2004) have previously been shown to have greater efficacy under normoxic, compared to hypoxic growth, conditions, a result also reported in the present paper. Preventing tumour hypoxia using darbepoetin not only increases the sensitivity of tumour cells to chemotherapy as the therapeutic index of most drugs is augmented under normoxic conditions, but also has the added benefit of slowing down the process of hypoxia-associated malignant progression. Moreover, the correction of anaemia and its associated symptoms, particularly fatigue, improves patients' quality of life. This may have implications for improving tolerance to therapy, which may lead to more patients completing an entire course of treatment with increased likelihood of therapeutic response (Blackwell *et al*, 2004).

We conclude that darbepoetin improves therapeutic outcome by an indirect mechanism, which is the prevention of anaemia and a subsequent reduction of tumour hypoxia, resulting in increased tumour cell chemosensitivity. A direct effect of darbepoetin on EpoR-expressing tumour cells is ruled out in this case, as the cells were shown not to respond to the hormone. We have also shown that darbepoetin treatment ‘normalises’ haemoglobin to control levels (Hb<14 g dl^−1^), not to suprapharmacological levels. This bodes well for clinical applications where elevated haemoglobin levels could have a negative effect due to thrombovascular risk. It must be noted that this is a single tumour model using a single treatment regimen, and further investigation into the consequences of darbepoetin treatment in combination with chemotherapy is necessary.

## Figures and Tables

**Figure 1:**
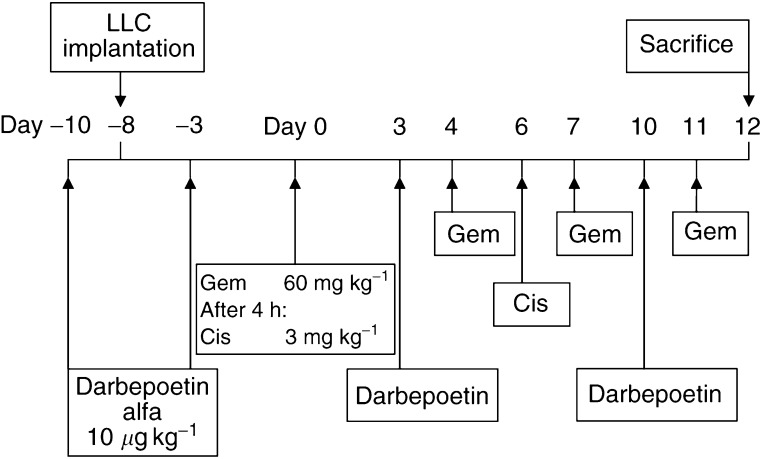
*In vivo* study schedule showing LLC implantation in C57 mice, darbepoetin treatment and metronomic dosing schedule of cisplatin (Cis) and gemcitabine (Gem).

**Figure 2:**
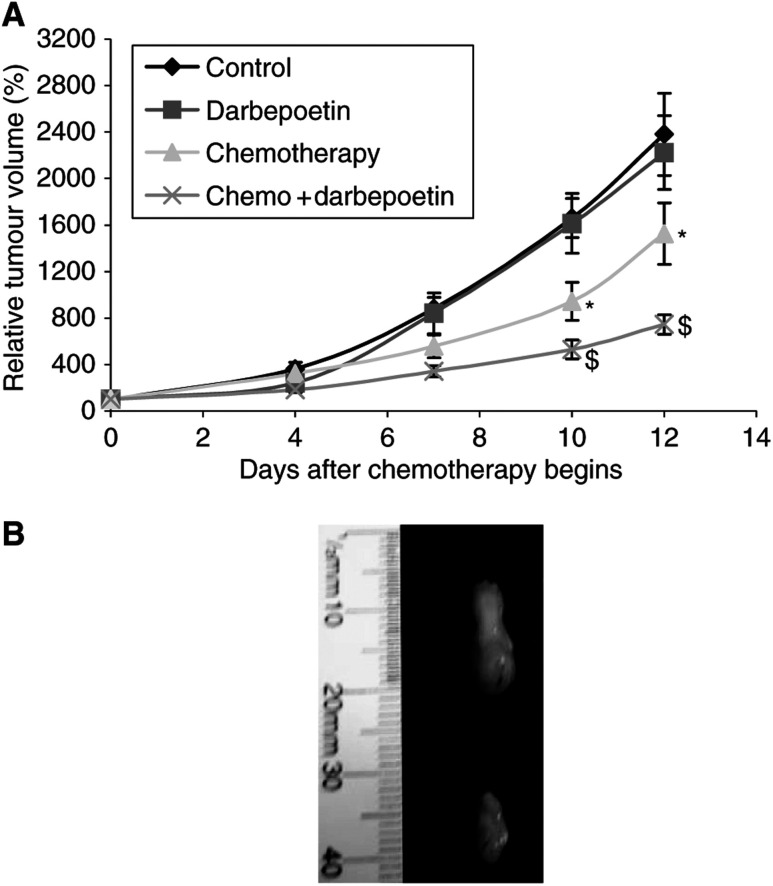
(**A**) Tumour growth curves for mice bearing flank tumours derived from LLC cells from the four treatment groups. Tumour volume is expressed relative to that determined on day 0 (100%). The average maximum tumour diameter on day 12 was 16 mm. Chemotherapy groups (^*^ and $) *P*<0.05 *vs* control; combination chemotherapy and darbepoetin ($) *P*<0.05 *vs* chemotherapy alone (^*^). Statistical analysis was performed by one-way ANOVA with LSD *post hoc* correction. Data are expressed as mean±s.e.m. (**B**) Photograph of representative tumours derived from LLC cells removed from chemotherapy and combination chemotherapy and darbepoetin-treated mice after the mice were killed on day 12 using cervical dislocation under anaesthetic. (i) Tumour from a mouse treated with chemotherapy only; (ii) tumour from a mouse treated with combination chemotherapy and darbepoetin.

**Figure 3:**
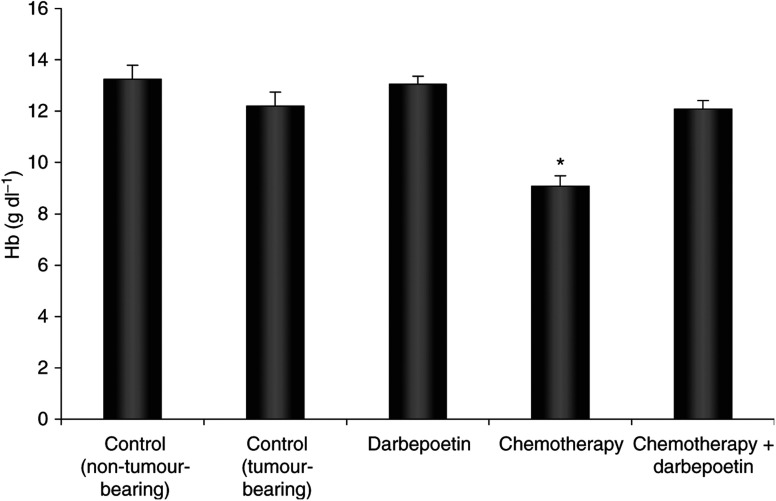
Blood haemoglobin concentration in the treatment groups, and in normal non-tumour-bearing mice. A blood sample was taken by carrying out a cardiac puncture while mice were anaesthetised. Blood was collected in a microcuvette, and haemoglobin measured using an automatic Hemocue Analyser. ^*^P<0.05 *vs* other groups (note that Hb<10 g dl^−1^ is indicative of anaemia). Data are expressed as mean±s.e.m.

**Figure 4:**
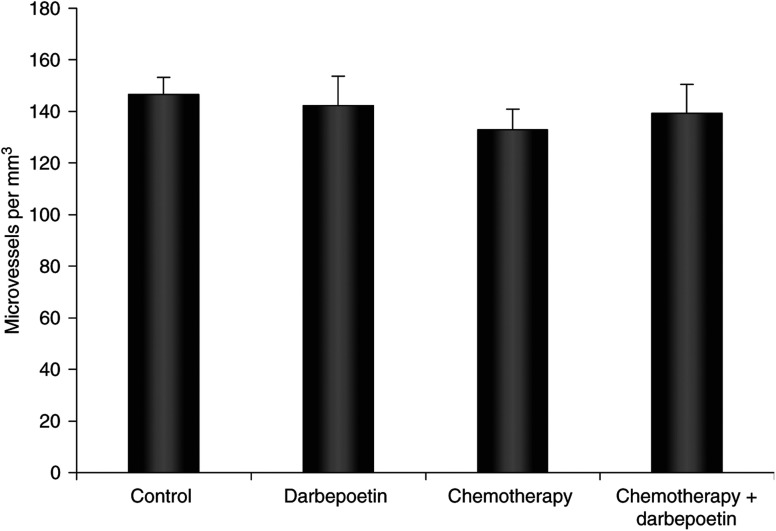
Tumour microvessel density, quantified by counting vessels stained with MECA32 panendothelial antigen. The data represent the number of microvessels per mm^3^ tumour area in the treatment groups (±s.e.m.).

**Figure 5:**
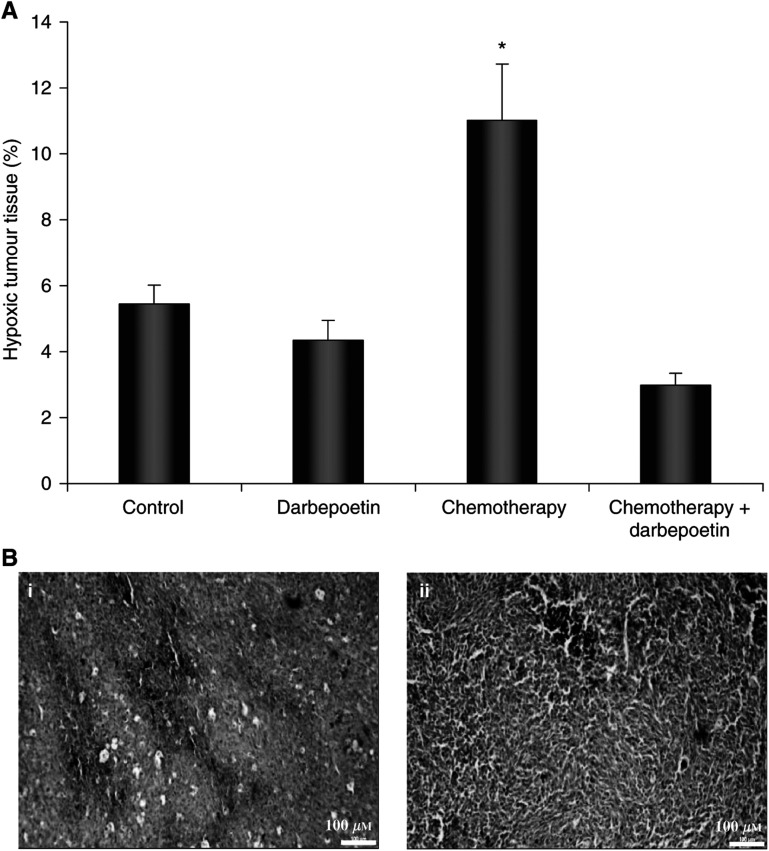
(**A**) Hypoxic tumour tissue, quantified by calculating Hypoxyprobe™-1 staining as a percentage of total tumour area. The data represent the mean percentage positive staining in the treatment groups (±s.e.m.). ^*^P<0.05 *vs* other groups. (**B**) Representative micrographs (× 100 magnification) are shown of Hypoxyprobe™-1 binding (brown staining) in (i) a chemotherapy-treated tumour and (ii) tumour treated with combination chemotherapy and darbepoetin.

**Figure 6:**
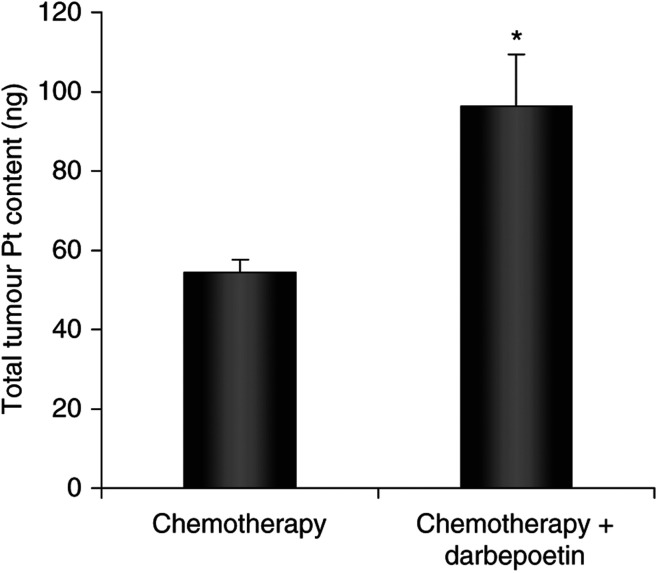
Tumour Pt content, measured using ICP-MS, in chemotherapy-treated tumours compared with tumours in the combination chemotherapy and darbepoetin group. Data are expressed as the mean±s.e.m. and statistical analysis was performed by an independent sample *t*-test. ^*^P<0.05 *vs* chemotherapy only.

**Figure 7:**
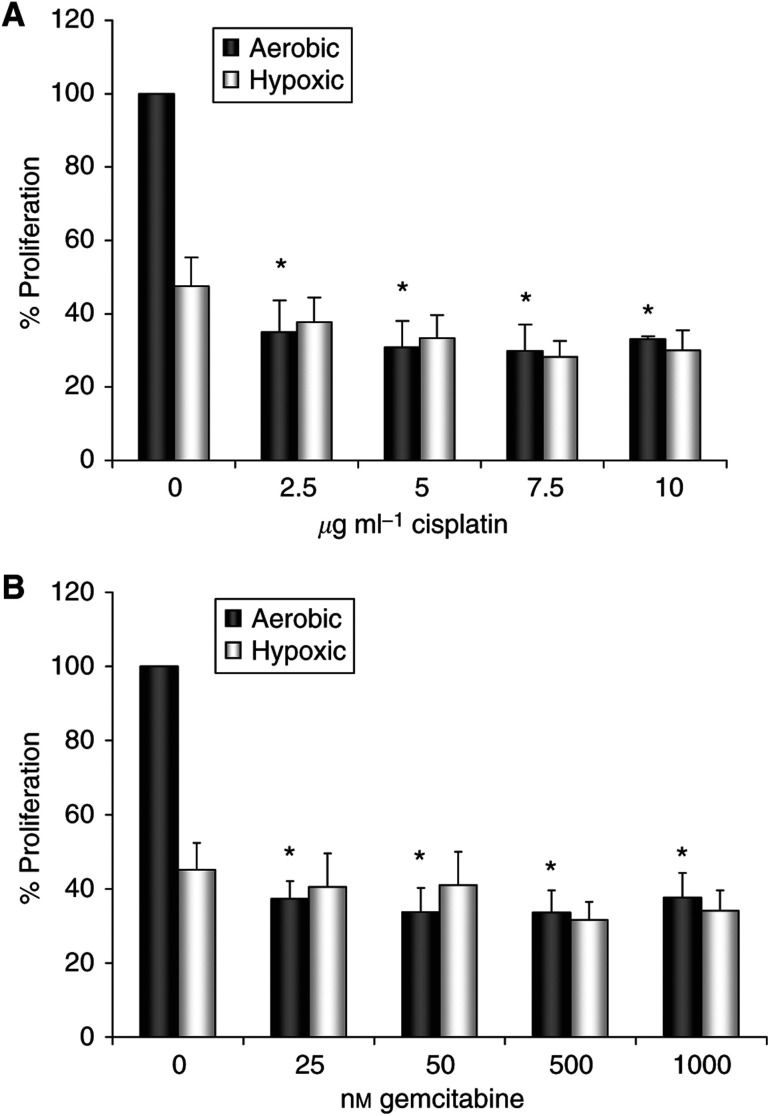
The cytotoxic effects of chemotherapies on tumour cell proliferation. Lewis lung carcinoma cells were cultured in the presence of cisplatin (**A**) and gemcitabine (**B**) and viability was assessed after 48 h under aerobic or hypoxic (1% O_2_) growth conditions using the MTT assay. Data are expressed as a percentage of untreated control cell proliferation under aerobic growth conditions (% means±s.e.m.). Data represent 3 independent 48 h MTT experiments. ^*^P<0.05 *vs* normoxic control.

**Figure 8:**
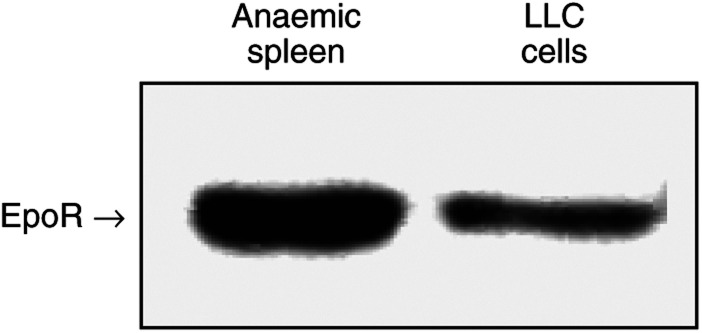
Lewis lung carcinoma cells were cultured under normal growth conditions and proteins were isolated from the cell line and from lysed murine anaemic spleens. Following separation of proteins by SDS–PAGE, proteins were transferred to a nitrocellulose membrane and probed with anti-EpoR antibody. EpoR expression was analysed by X-ray autoradiography. Data represent typical results of one of three independent experiments.

**Figure 9:**
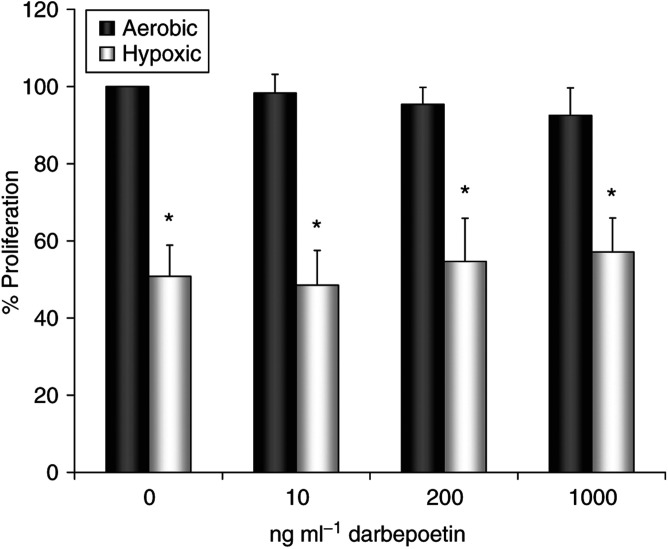
Lewis lung carcinoma cells were cultured in the presence of darbepoetin (10–1000 ng ml^−1^) under aerobic or hypoxic (1% O_2_) growth conditions, and proliferation was assessed after 48 h using the MTT assay. Hypoxic conditions significantly reduce LLC cell proliferation (^*^P<0.05 *vs* normoxic control). Data are expressed as a percentage of aerobic control (% means±s.e.m.) and represent three independent MTT experiments. Statistical analysis was performed by one-way ANOVA with LSD *post hoc* correction.

**Figure 10:**
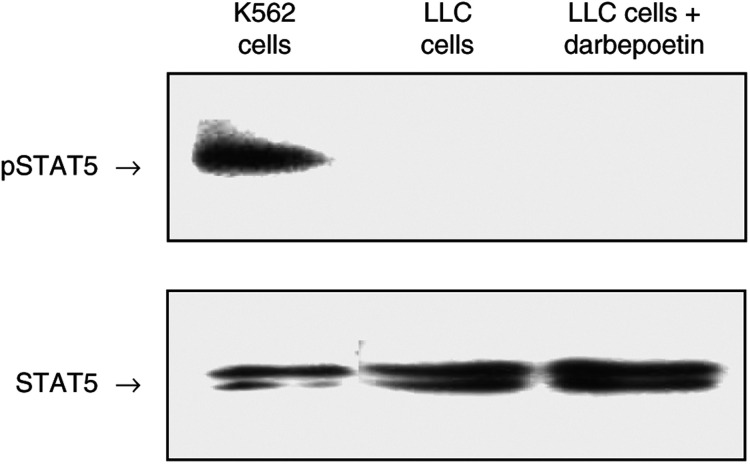
Lewis lung carcinoma cells were serum-starved for 5 h and then stimulated for 20 min with darbepoetin alfa (250 ng ml^−1^), or left untreated as a control. K562 cells, in which STAT5 is constitutively phosphorylated, were used as a positive control. After treatment, the cells were washed and lysed. Following separation of proteins by SDS–PAGE, blots were probed with anti-phospho-specific STAT5 antibody and pSTAT5 expression was analysed by X-ray autoradiography. Data represent typical results of one of three independent experiments. The membrane was stripped and probed with an antibody that recognises total STAT5 (bottom).
